# Tuning the Geometrical Structures and Optical Properties of Blue-Emitting Iridium(III) Complexes through Dimethylamine Substitutions: A Theoretical Study

**DOI:** 10.3390/molecules22050758

**Published:** 2017-05-07

**Authors:** Xue-Feng Ren, Hong-Qu Tang, Guo-Jun Kang

**Affiliations:** 1 Low Carbon Energy Institute, School of Chemical Engineering & Technology, China University of Mining &Technology, Xuzhou 221008, China; renxf@cumt.edu.cn (X.-F.R.); jzhangmt@126.com (H.-Q.T.); 2 Fukui Institute for Fundamental Chemistry, Kyoto University, 34-4 Takano Nishihiraki-cho, Sakyo, Kyoto 606-8103, Japan

**Keywords:** DFT, dimethylamine, phosphorescence, ^3^MC d-d excited states

## Abstract

The geometrical structures and photophysical properties of Ir(4,6-dFppy)_2_(pic) (FIrpic) and its derivative (*o*-FIr, *m*-FIr, *p*-FIr) with dimethylamine substituted at the picolinic acid (N^∧^O) ligand were fully investigated by density functional theory and time-dependent density functional theory. The simulated electronic structure, as well as absorption and emission spectra of FIrpic are in good agreement with the experimental observations. The introduction of dimethylamine at the N^∧^O ligand at different positions is beneficial to extend the π-electron delocalization, increase HOMO energy levels, and hence improve the hole injection and transfer ability compared with those of FIrpic. Furthermore, *o*-FIr, *m*-FIr, and *p*-FIr have large absorption intensity and participation of metal-to-ligand charge transfer (MLCT) contribution in the main absorption spectra, which would be useful to improve the intersystem crossing (ISC) from the singlet to triplet excited state. More importantly, the high quantum yield of *o*-FIr (which is explained based on the detailed analysis of triplet energy, E_T1_), participation of ^3^MLCT contribution in the phosphorescent spectra, and energy difference between ^3^MLCT and triplet metal centered (^3^MC) d-d excited state compared with *m*-FIr and *p*-FIr indicate that *o*-FIr is expected to be an excellent blue phosphorescence emitter with high efficiency.

## 1. Introduction

Phosphorescent transition metal materials such as Re(I), Ru(II), and Ir(III) complexes are of great interest [1,2,3,4], because these materials have been extensively investigated for optoelectronic and microelectronic applications, such as dye-sensitized solar cells (DSSCs) and organic light-emitting diodes (OLEDs) [5,6,7]. Among these emitters, Ir(4,6-dFppy)_2_(pic) (FIrpic) is one of the most well-known blue phosphorescent emitters, which is widely used as a dopant in the electroluminescent layers of OLEDs [8,9,10]. However, the quantum yield and equilibrium between the hole and electron transportation of FIrpic are considered to be difficult for its further application. The development of high-performance blue-emitting materials is strongly required for the fabrication of OLEDs. Unfortunately, it is a challenging task to achieve stable and high-efficiency blue phosphorescent emitters because the blue phosphorescence complex has a high emission state compared with other color complexes. This enables thermal activation to triplet metal centered (^3^MC) d-d state, which is one of the main nonradiative processes from the emissive excited state for Ir(III) complexes [11,12]. 

**Figure 1:**
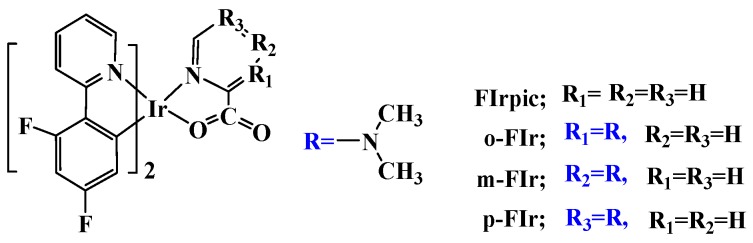
Schematic structures of the studied complexes.

In OLEDs, researchers can control the emission color and photophysical properties of emitters by a modification of the chelating ligands [13,14,15,16,17]. The common approach in tuning the photophysical properties of FIrpic is to modify the chemical structures of 2-(2,4-difluorophenyl) pyridine (C^∧^N) ligands. Baranoff et al. [18] replaced the F atoms on FIrpic by H, Cl, and Br. They found that the large halogen substituent contributed to sizable distortions of specific (C^∧^N) ligands, which were likely to play a role in the emissive and nonradiative properties when coupled with the heavy-atom effect. Owing to the introduction of the strong electron withdrawing perfluoro carbonyl group on (C^∧^N) ligands of FIrpic, the newly designed Ir complexes displayed deepest blue emissions and considerably high external quantum efficiencies (EQEs) [19]. Li et al. [20] investigated a series of Ir(III) complexes by introducing tert-butyl, *n*-heptyl, 3-ethylheptyl, and 2,4,6-trimethylphenyl substituted groups into the (C^∧^N) ligands of FIrpic. They revealed that both the substituted group and position had an effect on tuning the emission energies and quantum yield. However, the important chelating picolinic acid (N^∧^O) ligand of FIrpic has not received as much attention, to the best our knowledge. Herein, we will theoretically design and investigate a type of Ir complexes *o*-FIr, *m*-FIr, *p*-FIr (Figure 1) by introducing the dimethylamine substituted at the picolinic acid (N^∧^O) ligand, because the dimethylamine is one of the most popular functional groups used as hole transfer materials. We perform detailed theoretical calculations on the electronic structure, absorption and emission properties, quantum efficiency, as well as charge transport properties, aiming at revealing the relationship between the structures and photophysical properties. This work is expected to give some insights into the design and synthesis of blue emitters with high quantum efficiency.

## 2. Results and Discussion

### 2.1. The Optimized Geometries in the Ground and Lowest Lying Triplet Excited States 

**Figure 2:**
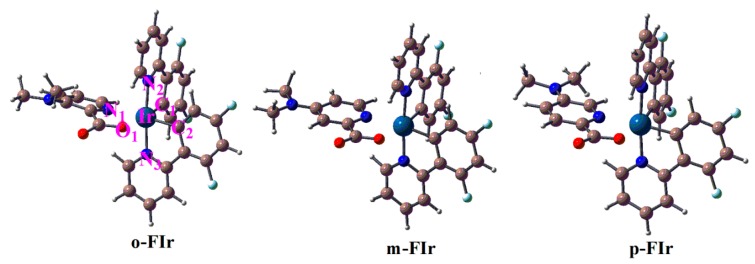
Optimized structures of *o*-FIr, *m*-FIr, and *p*-FIr in the ground state, together with the number of some key atoms.

The schematic structures of FIrpic, *o*-FIr, *m*-FIr, and *p*-FIr are depicted in Figure 1. The ground-state (S_0_) geometry of FIrpic was optimized by B3LYP, CAMB3LYP, M06L, MPW1PW91 functionals with the same basis set. The detailed geometries and available crystal data of FIrpic [21] are collected in Table S1 in supporting information. It is found that M06L functional predicts more satisfied geometrical structures by compared with the experimental data. Therefore, the S_0_ geometries of *o*-FIr, *m*-FIr, *p*-FIr are calculated by M06L functional, and the optimized geometries as well as the numbering of some key atoms are depicted in Figure 2. The optimized primary metal–ligand bond lengths of these designed complexes in the ground-state (S_0_) geometry are collected in Table 1.

As listed in Table 1, the N_1_-Ir-N_2_ and O_1_-Ir-N_2_ of FIrpic are ca. 90°, and the dihedral angle of N_1_-O_1_-C_2_-C_1_ of FIrpic is 6.8°. Obviously, FIrpic has distorted octahedral geometry around the Ir(Ш) atom. Table 1 shows that the N_1_-Ir-N_2_, O_1_-Ir-N_2_, and N_1_-O_1_-C_2_-C_1_ of *o*-FIr, *m*-FIr, and *p*-FIr are similar to those of FIrpic. Clearly, the introduction of a dimethylamine moiety on the pyridine moiety of picolinic acid (N^∧^O) does not affect the distorted octahedral geometry of the Ir(Ш) complex. For *o*-FIr and *p*-FIr, because of the introduction of dimethylamine group on the pyridine, the Ir-N_1_ and Ir-O_1_ of *o*-FIr and *p*-FIr are shortened (0.005 Å and 0.01 Å for *o*-FIr, while 0.05 Å and 0.016 Å for *p*-FIr) in comparison to those of FIrpic. These calculated results indicate that there is a strong interaction between the Ir(Ш) and (N^∧^O) ligand. Furthermore, the Ir-N_1_ bond length of *o*-FIr, *m*-FIr, and *p*-FIr are significantly shortened compared with that of FIrpic; therefore, the possible of dissociation of Ir-N_1_ bond in the ^3^MC state is expected to be decreased by the incorporation of N(CH_3_)_2_ groups.

The lowest lying triplet excited states (T_1_) of studied complexes are also listed in Table 1. As listed in Table 1, the Ir-N_1_ bond length of these complexes is dramatically elongated in the T_1_ state in comparison with that in S_0_ state. This suggests that the interaction between the N^∧^O ligand and the metal is weakened in the T_1_ state. From the S_0_ to the T_1_ state, the Ir-C_1/2_ and Ir-N_2/3_ bond lengths of 2-(2,4-difluorophenyl)pyridine (C^∧^N) ligands in *o*-FIr, *m*-FIr, and *p*-FIr are contracted by the substitutions of the dimethylamine groups on the pyridine moiety. The strengthened metal–ligand bonds will strength the interaction between the metal and C^∧^N ligands and further benefit the charge transfer from the metal to ligand, which may improve the quantum efficiency. 

### 2.2. Froniter Molecular Orbtials

**Figure 3:**
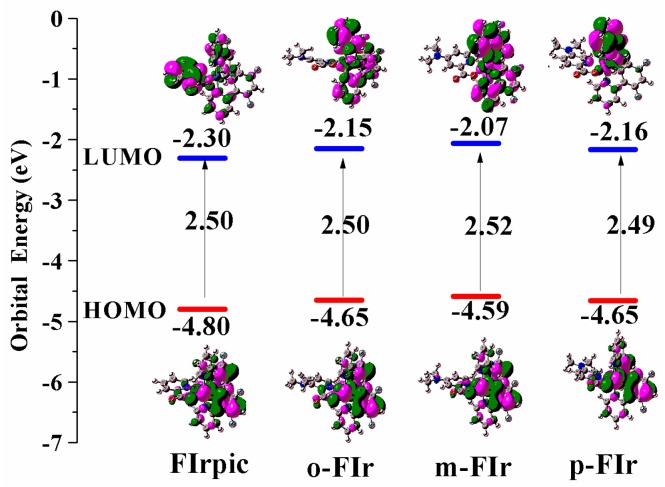
The orbital energies and contour plot of the HOMO and LUMO of studied complexes.

Considering that frontier molecular orbitals are useful to provide the nature of the charge transfer and photophysical properties in the excited state, the HOMO and LUMO energies, HOMO–LUMO energy gaps, and the contour plots of these orbitals of studied complexes are drawn in Figure 3. Detailed descriptions of the frontier molecular compositions are collected in Table S2 in the supporting information.

Normally, when the interfacial vacuum energy shift can be neglected, the hole (or electron) injection energy barrier from the electrode to semiconductor (Φ_h_) (or Φ_e_) can be calculated by Φ_h_ = |HOMO| − Φ_m_ (or Φ_e_ = Φ_m_ − |LUMO|), where Φ_m_ is the work function of the electrode [22]. Consider that Au (Φ_m_ = 5.1 eV) is widely used as electrode in OLEDs; therefore, it is used as reference electrode [22]. As depicted in Figure 3, the HOMO and LUMO energies of *o*-FIr, *p*-FIr, and *m*-FIr increase remarkably (−4.65 eV and −2.15 eV for *o*-FIr, −4.59 eV and −2.07 eV for *m*-FIr, −4.65 eV and −2.16 eV for *p*-FIr) compared with FIrpic (−4.80 eV and −2.30 eV). Therefore, the calculated Φ_h_ of these complexes are (in the region of 0.30–0.51 eV) much smaller than those of the calculated Φ_h_ values (in the region of 2.80–3.03 eV). It is obvious that these emitters are suitable for use as p-type organic semiconductors [23]. Furthermore, the HOMO energy level of *o*-FIr (0.15 eV), *m*-FIr (0.21 eV), and *p*-FIr (0.15 eV) is significantly higher than that of FIrpic. The higher HOMO energies will benefit the hole injection abilities. Together with the increasing HOMO and LUMO energy, the HOMO–LUMO energy gaps of our designed molecules are similar to that of FIrpic, which means that absorption and phosphorescent spectra of these designed complexes might be similar to that of FIrpic when these spectra are predominantly contributed by the HOMO→LUMO transition. 

For FIrpic, *o*-FIr, *p*-FIr, and *m*-FIr, the electron densities of HOMOs are mainly located on 50% d(Ir) orbital and 38% π(C^∧^N ligands) orbital, as shown in Figure 3 and Table S2. Owing to the introduction of N(CH_3_)_2_ groups, the LUMOs in *o*-FIr_,_ *m*-FIr_,_ and *p*-FIr are mainly focused on the C^∧^N ligands with about 90% compositions, while the LUMO in FIrpic is contributed by N^∧^O ligand. The HOMO-1 of these studied complexes is mainly focused on the ca. 50% d orbital the ca. 30% π orbitals of N^∧^O ligand, while theirs HOMO-2 are mainly contributed by N^∧^O ligand. The contributions of d(Ir) orbital on HOMO-3 of *o*-FIr (67.5%) and *m*-FIr (56.9%) are similar to that of FIrpic (67.1%). This means that the N(CH_3_)_2_ substitutions have a minor effect on the composition of the frontier molecular orbital. Therefore, the nature of electron transition upon excitations of *o*-FIr, *p*-FIr, and *m*-FIr might be similar with those of FIrpic, which will be further investigated in the following section.

### 2.3. Absorption Spectra

**Figure 4:**
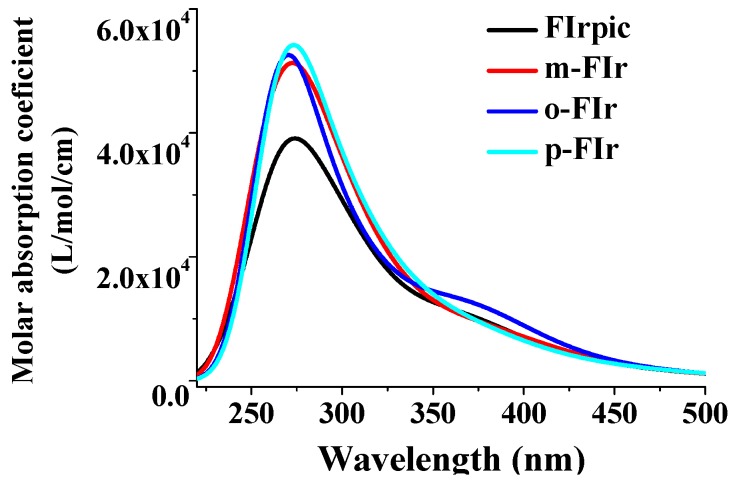
Simulated absorption spectra of studied complexes with the data calculated at the TD-M06L level.

The calculated absorption spectra of studied complexes associated with oscillator strengths (f) and the corresponding compositions, as well as the experimental data [24] are given in Table 2. Simulated absorption spectra of these complexes with the data calculated at the TD-M06L method are drawn in Figure 4. 

As listed in Table 2, the main calculated values of these bands are localized at 453.00, 380.95, and 265.86 nm for FIrpic, respectively, which are in good agreement with the experimental results of 458, 379, and 256 nm, respectively [24]. For the low-lying absorption spectra of FIrpic, the excitations of HOMO→LUMO + 1 and HOMO − 1→LUMO + 2 are dominantly responsible for the absorption peaks at 453.00 and 380.95 nm, respectively. As listed in Table S2, the HOMO and HOMO − 1 are delocalized on the Ir(d), N^∧^O, and N^∧^C ligand, while the LUMO + 1 and LUMO + 2 are mainly contributed by the N^∧^C ligand. Therefore, from the analysis of FMOs, the absorption peaks at 453.00 and 380.95 nm can be characterized as metal-to-ligand charge transfer (MLCT), inter-ligand charge transfer (ILCT), and ligand–ligand charge transfer (LLCT). The absorption peak with largest oscillator strength (*f* = 0.2104) of FIrpic is located at 265.86 nm, which is arising from the HOMO − 8→LUMO + 2 and HOMO→LUMO + 7 transitions. Because of the large of amount of Ir(d) in HOMO (50.7%) and HOMO – 8 (20.8%), the absorption peak at 265.86 nm can mainly be assigned to the MLCT/LLCT/ILCT character. From the analysis of frontier molecule orbital compositions, the mount of MLCT of the 453.00, 380.95, and 265.86 for FIrpic is 21.07%, 21.07%, and 7.16%, respectively. The significant MLCT contributions on the lower-lying absorptions of FIrpic are in good agreement with the experimental results [24]. 

Table 2 also shows the lowest-lying absorption peak of *o*-FIr, *m*-FIr, and *p*-FIr at 463.87, 462.95, and 463.29 nm, respectively, which is dominantly responsible for the excitation of HOMO→LUMO. Table S2 shows that the HOMO of *o*-FIr, *m*-FIr, and *p*-FIr is composed of 50.1% Ir(d) + 47.7% π(N^∧^O + N^∧^C), 50.3% Ir(d) + 47.9% π(N^∧^O + N^∧^C), 48.6% Ir(d) + 49.3% π(N^∧^O + N^∧^C), respectively, while their LUMOs are contributed by ca. 80% π*(N^∧^C). Thus, the lowest-lying absorptions of these complexes originate from the [Ir(d) + π(N^∧^O + N^∧^C)]→π*(N^∧^C) excited state with MLCT/ILCT/LLCT character. The mount of MLCT of the lowest-lying absorption of *o*-FIr, *m*-FIr, and *p*-FIr is ca.22%, which is similar to that of FIrpic. Furthermore, as shown in Figure 4, the absorption intensity of N(CH_3_)_2_-substituted Ir complexes (*m*-FIr, *o*-FIr, *p*-FIr) in the highest energy absorption spectra have been effectively enhanced, which may increase the intersystem crossing rates (ISC). For *o*-FIr, the highest energy absorption peak (262.33 nm) is contributed by the mixture excitations of HOMO − 9→LUMO + 2, HOMO − 5→LUMO + 5, HOMO − 4→LUMO + 5, HOMO − 2→LUMO + 5, HOMO − 1→ LUMO + 5. Since the Ir(d) in the HOMO − 9, HOMO − 4, HOMO is 22.7%, 67.5%, 50.1%, respectively, the MLCT contributed 8.9% to the 262.33 nm of *o*-FIr, which is slightly larger than that of FIrpic. For *m*-FIr and *p*-FIr, the absorption peak with large oscillator strength is located at 267.06 (attributed by HOMO − 8→LUMO + 2 and HOMO→LUMO + 7) and 271.08 nm (attributed by HOMO − 7→LUMO + 4, HOMO − 3→LUMO + 5, HOMO − 2→LUMO + 5, HOMO→LUMO + 7), respectively. Combining the transition configuration with the orbital composition, the mount of MLCT of the high energy absorption peaks for *m*-FIr and *p*-FIr are only 4.4% and 5.2%, respectively. Clearly, the contribution of MLCT in the whole absorption spectra of *o*-FIr are remarkably larger than those of FIrpic, *m*-FIr, and *p*-FIr. Normally, the participation of MLCT in the absorption spectra is a critical factor affecting the intensity of the singlet–triplet transitions, and thus improves the quantum efficiency. Thus, the calculated results indicate that the introduction of N(CH_3_)_2_ groups at the ortho-position of N^∧^O ligand is useful to enhance the intersystem crossing and singlet–triplet transitions, and therefore the possibility of the phosphorescent quantum yield might be enhanced.

### 2.4. Phosphorescence Emission Spectra

Based on the optimized lowest triplet excited state (T_1_) geometries, vertical T_1_–S_0_ transition energies (ΔE_vert_) and the emission wavelength (nm) obtained by CAMB3LYP functional are collected in Table 3, together with available experimental values [24]. Since the vertical T_1_–S_0_ transition energy (ΔE_vert_) is the electronic energy difference between the T_1_ and S_0_ state at the optimized T_1_ geometry, it is therefore strongly dependent on the functional. The molecular orbital compositions responsible for the emission spectra of the studied complex are given in Table S3. The 0-0 T_1_→S_0_ transition energy (ΔE_0-0_) is calculated by the energy difference between the S_0_ and T_1_ states at their respective optimized geometries obtained by M06 functional. 

As listed in Table 3, the calculated emission peak for FIrpic based on the calculated E_vert_ and E_0-0_ method is located at 490.49 and 480.86 nm, respectively. The slightly longer wavelength derived from E_vert_ is caused by only one single configuration, while real emission is composed of more than one configuration. The phosphorescence at 490.49 nm of FIrpic is contributed by the excitation of HOMO→LUMO. According to the orbital composition analyses, the HOMO is contributed by 42.8% Ir(d) + 54.3% π(C^∧^N + N^∧^O), while the LUMO is mainly focused on C^∧^N ligand. Accordingly, the emission of FIrpic can be attributed to ^3^MLCT/^3^LLCT/^3^ILCT character. The d(Ir) orbital contributes to 42.8% for HOMO, and thus the emission of FIrpic at 490.49 nm has ^3^MLCT (20.31%) transition character. For N(CH_3_)_2_-substituted Ir complexes, the phosphorescent emissions based on E_vert_ method are located at 492.78, 484.32, and 485.83 nm for *o*-FIr, *m*-FIr, and *p*-FIr, respectively, which is dominantly responsible for excitations of HOMO→LUMO and HOMO − 4→LUMO. This is because the HOMO and HOMO − 4 of *o*-FIr, *m*-FIr, and *p*-FIr are mainly contributed by d(Ir) and π(C^∧^N + N^∧^O) (besides the HOMO − 4 of *m*-FIr), while the LUMOs of *o*-FIr and *m*-FIr are located at π*(C^∧^N). Table 3 shows that the calculated E_0–0_ for these designed complexes are ca. 480nm. Accordingly, these designed complexes have similar phosphorescent emissions and transition character with that of FIrpic, which might be a potential candidate for blue-emitting materials. Furthermore, the d(Ir) orbital contributions to HOMO and HOMO − 4 (42.8% and 0.97% for *m*-FIr, 30.68% and 36.68% for *p*-FIr) are much smaller than that of HOMO (41.35%) and HOMO − 4 (63.39%) for *o*-FIr. The larger d(Ir) orbital composition results in slightly larger ^3^MLCT% in its phosphorescence of *o*-FIr (18.78%) than *m*-FIr (9.11%) and *p*-FIr (11.56%). Therefore, the *o*-FIr may have improved Φ_p_ compared with *m*-FIr and *p*-FIr. 

**Figure 5:**
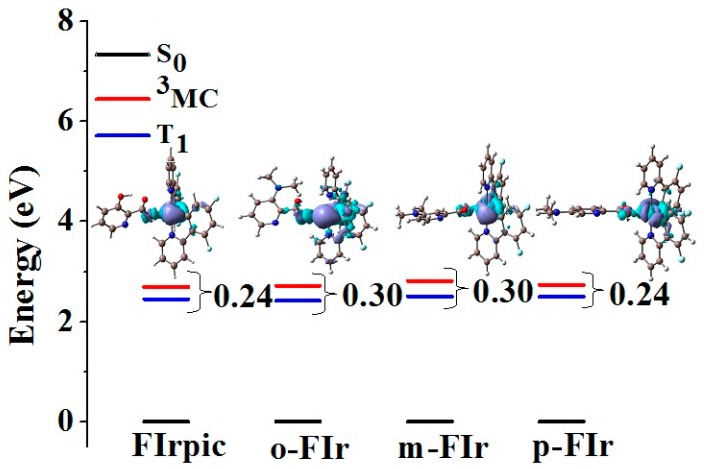
Energy level diagram of the studied complexes in the T_1_ and triplet metal centered (^3^MC) excited states, together with the contour plots of the spin density distribution in the ^3^MC state.

To give some insight into the nonradiative rate constant (k_nr_), the high lying metal-centered (^3^MC d-d) excited states of studied complexes—which is one of the most important deactivation pathways for the phosphoresce emission from T_1_ in transition-metal complexes—were optimized by unrestricted M06L method. The energy difference between ^3^MLCT/π-π* and ^3^MC d-d excited states are drawn in Figure 5, along with the spin density distribution in the ^3^MC d–d state. As shown in Figure 5, the ^3^MC d-d state of studied complexes are lying at a relatively higher energy than that of ^3^MLCT/π-π* state, which suggests that the possibility of a radiationless pathway is low. Furthermore, the energy difference between the ^3^MLCT/π-π* and ^3^MC d-d excited states of *o*-FIr (0.30 eV) and *m*-FIr (0.30 eV) is larger than that of FIrpic (0.24 eV). Therefore, these calculation results show that ortho- and meta- substituted Ir complexes are useful to decrease the K_nr_. Based on the above viewpoint, it becomes clear that introducing the N(CH_3_)_2_ at the ortho-position of N^∧^O ligand in FIrpic led to larger ISC and k_r_ values, as well as the smaller k_nr_ value relative to others. Therefore, *o*-FIr is considered to be used as potential blue-emitting emits with high quantum efficiency.

### 2.5. Performance in OLEDs

To evaluate the performance of OLED devices, it is highly important to investigate the charge injection and transfer ability. The ionization potential (IP) and electron affinity (EA) are usually employed to evaluate the energy barrier for the hole and electron injections. Normally, the lower IP (larger EA) value suggests that it is easier to inject hole (electron). According to the Marcus theory [25], the electron transfer rate constants can be expressed by the following formula:

(1) $$k={\left(\frac{\pi }{\lambda k_{B}T}\right)}^{1/2}\frac{V^{2}}{\hbar }\exp(-\frac{\lambda }{4k_{B}T})$$

where T and k_B_ is the temperature and Boltzmann constant, respectively. The λ is the reorganization energy, and V is the coupling matrix element. According to Equation (1), the intermolecular charge transfer rate k is determined by two factors, λ and V. Due to the limited intermolecular charge transfer range in the solid state, the mobility of charges has been demonstrated to be primarily associated with the reorganization energy λ for OLED materials [26]. The detailed description of the calculations of vertical ionization potential (IP(v)), vertical electron affinity (EA(v)), hole extraction potential, (HEP), electron extraction potential (EEP) and electron reorganization energy (λ_electron_), as well as hole reorganization energy (λ_hole_) are listed in our previous report [16]. According to the previous report [16], the IP, EA, and λ of studied complexes are calculated and listed in Table 4.

Compared with FIrpic, all these designed complexes have smaller IP(v) and HEP, which is consistent with their higher HOMO energy levels. Thus, the hole injection ability of these designed complexes are improved by introduction of dimethylamine substitutions at the C^∧^O ligand. However, *o*-FIr, *m*-FIr, and *p*-FIr have smaller EA values than FIrpic, which will lead to worse electron injection ability compared with FIrpic. According to Equation (1), the λ is required to be small for efficient charge transport. As listed in Table 4, the λ_electron_ of studied complexes are slightly smaller than that of FIrpic, indicating that these complexes have excellent electron transfer ability. The difference between the λ_hole_ and λ_electron_ of FIrpic, *o*-FIr, and *p*-FIr is less than 0.05 eV. Hence, it is expected that the FIrpic, *o*-FIr, and *p*-FIr might have excellent charge transfer balance, which is one of the key factors affecting the performance of OLEDs.

## 3. Computational Details

The geometrical structures of the ground state (S_0_) of FIrpic were fully optimized by B3LYP [27,28], CAMB3LYP [29], M06L [30], and mPW1PW91 [31] methods, respectively, combined with 6-31G(d) basis set for the non-metal atoms (H, C, N, O, and F) and a “double-j” quality basis set LANL2DZ for Ir atom. The calculated vibrational frequencies with no imaginary frequencies based on the optimized geometries verify that each of the optimized structures was a true minimum on the potential energy surface. The calculated results are collected in Table S1 in the supporting information, together with the crystal data [21]. As depicted in Table S1, the bond lengths obtained for B3LYP and CAMBLYP overestimated the bond lengths, especially Ir-N_1_ (0.024 Å for CAMB3LYP and 0.031 Å for B3LYP) and Ir-N_3_ (0.049 Å for CAMB3LYP and 0.073 Å for B3LYP), while the bond length differences between the calculated results obtained by M06L and crystal data were small. Therefore, the ground-state geometries of the studied complexes were calculated by M06L method. Based on the ground-optimized structure, the vertical excitation energies were calculated by TD-M06L method with continuum polarizable continuum model (CPCM). The lowest-lying triplet excited state geometries (T_1_) and triplet-centered (^3^MC) d-d states were optimized by unrestricted M06L method. Based on the T_1_ equilibrium geometries, four different functionals—PBE0, B3LYP, CAMB3LYP, and M06L—were performed on the emission spectra calculations (as listed in Table S4). The CAMB3LYP functional was adopted to predict the emission spectra of these complexes, because the calculated results obtained by CAMB3LYP were consistent with the experimental results. All calculations were performed with the Gaussian 09 software package [32].

## 4. Conclusions

In this work, the geometrical and electronic structures, absorption and emission spectra, as well as phosphorescence efficiencies for a series of dimethylamine-substituted iridium(Ш) complexes FIrpic have been investigated by density functional theory (DFT) and time-dependent density functional theory (TDDFT) methods. The results indicate that the introduction of dimethylamine is useful to extend the π-electron delocalization, increase HOMO energy levels, and enhance hole injection and transfer ability. Furthermore, the absorption intensity and participation of MLCT of these dimethylamine substitutions are significantly stronger than that of FIrpic, suggesting the possibility of singlet-to-triplet transitions are enhanced, and therefore the phosphorescent quantum yield might be increased by introducing dimethylamine. Importantly, through the analyses of triplet energy (E_T1_), participation of ^3^MLCT in phosphorescent spectra, as well as the energy difference between the ^3^MLCT/π-π and ^3^MC d-d state, it can be inferred that *o*-FIr has larger k_r_ values and smaller k_nr_ values compared with other molecules. So, the strategy of introducing the dimethylamine in N^∧^O ligand of FIrpic should be useful in the design of more-efficient blue emitters. 

## Figures and Tables

**Table 1:** Calculated metal–ligand bond lengths (Å), bond angles (°), and dihedral angles (°) for studied complexes in the ground state (S_0_) and first excited triplet state(T_1_).

Molecule	State	Ir-N_1_	Ir-O_1_	Ir-N_2_	Ir-C_1_	Ir-N_3_	Ir-C_2_	N_1_-Ir-N_2_	O_1_-Ir-N_2_	N_1_-O_1_-C_2_-C_1_
FIrpic	S_0_	2.215	2.190	2.066	2.001	2.058	1.997	88.4	93.5	6.8
	T_1_	2.313	2.172	2.069	1.979	2.060	1.975	97.1	88.7	−3.4
*o*-FIr	S_0_	2.210	2.180	2.066	2.002	2.054	1.997	87.3	94.0	5.0
	T_1_	2.316	2.154	2.057	1.981	2.067	1.977	84.4	93.7	3.1
*m*-FIr	S_0_	2.206	2.193	2.064	2.002	2.056	1.995	88.6	93.0	7.2
	T_1_	2.285	2.171	2.069	1.979	2.057	1.978	95.9	89.0	−4.6
*p*-FIr	S_0_	2.165	2.177	2.055	2.010	2.045	2.001	87.6	92.9	6.3
	T_1_	2.305	2.160	2.068	1.982	2.058	1.977	97.4	88.7	−3.0

**Table 2:** The calculated maximum absorption wavelength (nm), oscillator strengths (*f*), and major contribution transition composition for studied complexes obtained by TD-M06L method. FIrpic: Ir(4,6-dFppy)_2_(pic).

Molecule	Transition	λ(nm)	*f*	Composition	CI	Exp. [24] (nm)
FIrpic	S_0_→S_2_	453.00	0.0250	HOMO→LUMO + 1	0.68971	455
	S_0_→S_7_	380.95	0.0614	HOMO − 1→LUMO + 2	0.55936	379
	S_0_→S_50_	265.86	0.2104	HOMO − 8→LUMO + 2	0.38300	256
				HOMO→LUMO + 7	0.24359	
*o*-FIr	S_0_→S_1_	463.87	0.0258	HOMO→LUMO	0.70168	
	S_0_→S_7_	395.15	0.0266	HOMO − 2→LUMO + 2	0.56244	
				HOMO − 1→LUMO + 2	−0.40890	
	S_0_→S_52_	269.04	0.1135	HOMO − 9→LUMO + 1	0.25480	
				HOMO − 5→LUMO + 5	0.42176	
				HOMO − 1→LUMO + 7	0.23321	
	S_0_→S_53_	268.66	0.1642	HOMO − 7→LUMO + 4	−0.35667	
				HOMO − 1→LUMO + 7	0.36355	
	S_0_→S_54_	266.76	0.1318	HOMO − 9→LUMO + 2	0.21920	
				HOMO − 2→LUMO + 7	0.26962	
				HOMO − 1→LUMO + 7	−0.21838	
				HOMO→LUMO + 7	0.41903	
	S_0_→S_55_	266.32	0.0836	HOMO − 5→LUMO + 5	0.42068	
	S_0_→S_56_	264.54	0.1094	HOMO − 9→LUMO + 2	0.48856	
				HOMO − 8→LUMO + 2	−0.19242	
				HOMO − 1→LUMO + 5	0.27865	
	S_0_→S_57_	262.33	0.1716	HOMO − 9→LUMO + 2	−0.24371	
				HOMO − 5→LUMO + 5	−0.24091	
				HOMO − 4→LUMO + 5	−0.25407	
				HOMO − 2→LUMO + 5	0.20626	
				HOMO − 2→LUMO + 7	0.30782	
				HOMO − 1→LUMO + 5	0.32002	
*m*-FIr	S_0_→S_1_	462.95	0.0263	HOMO→LUMO	0.70166	
	S_0_→S_6_	393.55	0.0423	HOMO − 2→LUMO	−0.41398	
				HOMO − 2→LUMO + 1	0.42182	
				HOMO − 1→LUMO + 1	0.31148	
	S_0_→S_53_	267.06	0.3060	HOMO − 8→LUMO + 2	−0.24031	
				HOMO→LUMO+7	0.25154	
*p*-FIr	S_0_→S_1_	463.29	0.0286	HOMO→LUMO	0.70148	
	S_0_→S_8_	388.02	0.0581	HOMO − 1→LUMO + 1	−0.43685	
				HOMO→LUMO + 3	0.47613	
	S_0_→S_52_	271.08	0.2512	HOMO − 7→LUMO + 4	−0.27219	
				HOMO − 3→LUMO + 5	0.21898	
				HOMO − 2→LUMO + 5	0.29024	
				HOMO→LUMO + 7	−0.21723	

**Table 3:** The calculated phosphorescence emission wavelength (nm), along with metal–ligand charge transfer character (^3^MLCT, %) and available experimental values.

Molecule	E_vert_				E_0-0_		Exp [24]
	λ (nm)	E (eV)	Composition	^3^MLCT (%)	λ (nm)	E (eV)	λ (nm)
FIrpic	490.49	2.53	0.49 (HOMO→LUMO)	20.31	480.86	2.58	468
*o*-FIr	492.78	2.52	0.45 (HOMO − 4→LUMO) 0.43 (HOMO→LUMO)	18.78	482.58	2.60	
*m*-FIr	484.32	2.56	−0.36 (HOMO − 4→LUMO) 0.40 (HOMO→LUMO)	9.11	481.78	2.57	
*p*-FIr	485.83	2.55	−0.36 (HOMO − 4→LUMO) 0.49 (HOMO→LUMO)	11.56	481.76	2.57	

**Table 4:** The calculated ionization potential (IP, eV), electron affinities (EA, eV), hole extraction potential, (HEP, eV), electron extraction potential (EEP, eV) hole/electron reorganization energy (λ_hole_/λ_electron_, eV).

Molecule	IP(v)	HEP	EA(v)	EEP	λ_hole_	λ_electron_
FIrpic	6.56	6.29	0.72	0.98	0.27	0.26
*o*-FIr	6.32	6.06	0.48	0.69	0.26	0.21
*m*-FIr	6.39	6.09	0.56	0.75	0.30	0.19
*p*-FIr	6.34	6.11	0.53	0.73	0.23	0.20

## Supplementary Materials

Supplementary materials are available on line.
