# Supplementary material for: Tuning the Geometrical Structures and Optical Properties of Blue-Emitting Iridium(III) Complexes through Dimethylamine Substitutions: A Theoretical Study
Source: Molecules. 2017 May 7;22(5):758. doi: 10.3390/molecules22050758 (PMC6153745; doi:10.3390/molecules22050758)
Supplement: Supplementary file 1 [file molecules-22-00758-s001.pdf]

Supporting Information

Tuning the Geometrical Structures and Optical Properties of Blue-Emitting Iridium(III) Complexes Through Dimethylamine Moiety substitutions: A Theoretical Study

Xue-Feng Ren[1,2] Hong-Qu Tang[1] Guo-Jun Kang[1] *

([1]Low Carbon Energy Institute, School of Chemical Engineering & Technology, China University of Mining &Technology, Xuzhou 221008, China)

(2 Fukui Institute for Fundamental Chemistry, Kyoto University, 34-4 Takano Nishihiraki-cho, Sakyo, Kyoto 606-8103, Japan)

* Correspondence: gjkang@cumt.edu.cn

Table S1. Calculated metal ligand bond lengths (Å), and bond angles (°), and dihedral angles (°) for studied complexes in the ground state (S_0) and first excited triplet state(T_1).

Method	Ir-N_1	Ir-O_1	Ir-N_2	Ir-C_1	Ir-N_3	Ir-C_2
B3LYP	2.211	2.180	2.062	2.011	2.072	2.013
CAMB3LYP	2.160	2.187	2.065	2.006	2.052	2.004
M06L	2.215	2.190	2.066	2.001	2.058	1.997
MPW1PW91	2.173	2.157	2.039	1.993	2.050	1.998
crystal[a]	2.126	2.174	2.043	1.996	2.023	1.991

[a]Acta Cryst. (2009). E65, m28

Table S2. The molecular orbital composition (%) in the ground state of studied complexes.

Complex	Orbital	Energy	MO contribution (%)			
		(eV)	Ir(d)	N^O	N^C_1	N^C_2
**FIrpic**	H-8	-6.43	20.8	61.3	7.0	8.8
	H-7	-6.23	2.5	60.2	20.0	13.9
	H-6	-6.05	3.0	5.8	53.0	37.6
	H-5	-5.82	11.6	2.6	76.3	9.3
	H-4	-5.65	3.0	5.7	3.4	87.8
	H-3	-5.51	67.1	5.3	19.2	7.7
	H-2	-5.33	2.6	87.7	5.4	3.5
	H-1	-5.11	54.7	25.4	8.4	10.2
	H	-4.80	50.7	9.4	18.7	19.5
	L	-2.30	0.6	79.7	13.4	5.1
	L+1	-2.22	6.4	16.7	71.7	4.4
	L+2	-2.13	5.5	3.1	7.3	83.3
	L+3	-1.85	0.1	66.6	20.3	11.8
	L+4	-1.63	1.1	17.1	76.8	4.1

	L+5	-1.57	0.7	14.4	1.1	83.2
	L+6	-0.66	6.3	7.5	64.7	19.7
	L+7	-0.39	22.2	4.0	24.8	47.7
**o-FIr**	H-10	-6.92	6.0	32.5	3.9	53.2
	H-9	-6.27	22.7	58.1	8.1	9.1
	H-8	-6.14	3.2	59.4	17.6	16.7
	H-7	-5.94	3.1	6.1	55.8	34.5
	H-6	-5.72	8.6	2.3	77.2	11.7
	H-5	-5.53	2.3	5.4	4.0	88.3
	H-4	-5.36	67.5	8.1	16.5	7.1
	H-3	-5.21	7.2	84.4	4.9	2.7
	H-2	-5.04	1.5	92.8	2.3	3.0
	H-1	-4.91	49.5	32.7	8.2	8.3
	H	-4.65	50.1	10.2	18.1	19.4
	L	-2.15	4.8	2.8	85.2	6.1
	L+1	-1.99	4.9	22.0	5.9	66.3
	L+2	-1.97	2.9	72.9	4.0	19.5
	L+3	-1.62	0.5	6.7	83.3	8.7
	L+4	-1.46	0.8	2.6	9.5	86.3
	L+5	-1.09	0.6	92.0	2.3	3.9
	L+6	-0.52	5.2	7.4	66.8	18.8
	L+7	-0.26	19.7	4.0	24.4	50.8
**m-FIr**	H-8	-6.03	3.3	58.9	18.1	16.3
	H-7	-5.89	4.0	9.1	54.1	32.1
	H-6	-5.81	19.2	60.1	11.0	9.4
	H-5	-5.62	0.4	17.6	76.1	5.6
	H-4	-5.48	2.0	5.7	3.3	88.9
	H-3	-5.15	56.9	26.6	9.6	6.3
	H-2	-5.10	3.5	87.4	5.3	2.9
	H-1	-4.90	53.5	26.9	8.4	9.9
	H	-4.59	50.3	10.4	18.4	19.1
	L	-2.07	4.6	3.0	83.6	7.7
	L+1	-1.96	5.5	1.7	8.6	83.4
	L+2	-1.68	1.6	75.6	13.1	9.1
	L+3	-1.51	0.1	27.8	63.1	8.0
	L+4	-1.43	0.9	5.5	18.0	74.9
	L+5	-1.24	1.3	85.7	4.2	7.8
	L+6	-0.49	5.2	6.7	67.0	19.5
	L+7	-0.21	19.7	3.9	24.7	50.4
**p-FIr**	H-7	-5.99	3.1	4.4	51.3	40.5
	H-6	-5.78	9.4	5.8	74.4	10.1
	H-5	-5.53	1.8	5.6	3.3	89.2
	H-4	-5.45	39.8	39.9	14.7	5.0
	H-3	-5.30	31.9	59.6	4.5	3.5

H-2	-5.04	7.4	84.3	5.1	2.3
H-1	-4.93	46.6	34.3	8.4	9.2
H	-4.65	48.6	11.8	17.9	19.6
L	-2.16	4.3	3.2	86.1	5.2
L+1	-2.01	5.4	2.8	6.3	84.6
L+2	-1.87	2.5	91.8	1.2	3.1
L+3	-1.64	1.3	8.3	78.2	11.2
L+4	-1.48	0.8	2.9	14.2	81.4
L+5	-1.21	0.1	89.5	4.4	5.6
L+6	-0.60	4.9	6.2	74.6	12.7
L+7	-0.29	19.8	4.7	18.4	54.1

Table S3. The molecular orbital composition (%) in the triplet state of studied complexes.

Complex	Orbital	MO contribution (%)			
		Ir(d)	N^O	N^C1	N^C2
**FIrpic**	H-3	62.85	8.55	16.75	10.90
	H-2	2.88	88.48	5.70	2.18
	H-1	57.24	26.52	8.01	7.34
	H	42.79	15.25	18.36	20.64
	L	4.44	3.03	85.89	5.42
	L+6	8.90	8.54	66.59	14.00
**o-FIr**	H-4	62.39	10.08	15.40	11.17
	H	41.35	16.81	18.25	20.33
	L	4.99	3.57	85.73	4.60
	L+6	8.54	9.59	66.78	12.98
**m-FIr**	H-4	0.97	6.02	2.5	90.37
	H-3	40.9	44.99	8.1	5.07
	H-2	16.94	71.15	7.34	3.93
	H-1	57.45	26.5	7.96	7.16
	H	42.8	15.55	18.38	20.29
	L	4.54	2.81	86.68	4.87
**p-FIr**	H-4	30.68	27.13	8.01	12.13
	H-2	-1.65	92.11	4.01	1.35
	H	36.68	19.38	18.35	18.79
	L	2.293	58.12	22.56	13.35

Table S4 The calculated emission spectra (nm) obtained by different functional, along with the experimental data.

M06l	B3LYP	M062X	CAMB3LYP	PBE0	Exp.[24]
617.98	543.16	438.33	490.49	529.64	468
